# Knowledge regarding human monkeypox among a sample of undergraduate and post-graduate students from selected tertiary institutions in Bangladesh: An online-based cross-sectional study

**DOI:** 10.1371/journal.pone.0315677

**Published:** 2024-12-31

**Authors:** Md. Hasan Al Banna, Abdul-Aziz Seidu, Trisha Mallick, Nahidur Rahman, Mst. Sadia Sultana, Humayra Alam Mouly, Najim Z. Alshahrani, Nargees Akter, Tareq Mahmud, Susmita Hossain, Anannya Sheikh, Ashish Biswas, Sumaia Sahrin, Md. Nazmul Hassan, Md. Shafiqul Islam Khan

**Affiliations:** 1 Department of Food Microbiology, Faculty of Nutrition and Food Science, Patuakhali Science and Technology University, Patuakhali, Bangladesh; 2 Public Health and Tropical Medicine, James Cook University, Townsville, Australia; 3 Department of Environmental Sanitation, Faculty of Nutrition and Food Science, Patuakhali Science and Technology University, Patuakhali, Bangladesh; 4 Department of Food Processing and Engineering, Chattogram Veterinary and Animal Sciences University, Chattogram, Bangladesh; 5 Department of Health, Society, & Behavior, UC Irvine Joe C. Wen School of Population & Public Health, Irvine, California, United States of America; 6 Department of Public Health, North South University, Dhaka, Bangladesh; 7 Department of Family and Community Medicine, Faculty of Medicine, University of Jeddah, Jeddah, Saudi Arabia; 8 Department of Geography and Environmental Studies, University of Chittagong, Chittagong, Bangladesh; 9 Department of Public Health and Informatics, Jahangirnagar University, Dhaka, Bangladesh; 10 Bangladesh Dental College and Hospital, Dhaka, Bangladesh; 11 Colonel Maleque Medical College, Manikganj, Bangladesh; 12 Faculty of Agriculture, Patuakhali Science and Technology University, Patuakhali, Bangladesh; 13 Department of Human Nutrition and Dietetics, Faculty of Nutrition and Food Science, Patuakhali Science and Technology University, Patuakhali, Bangladesh; 14 Department of Cellular and Molecular Biology, Faculty of Biotechnology and Genetic Engineering, Chattogram Veterinary and Animal Sciences University, Chattogram, Bangladesh; Virginia Commonwealth University, UNITED STATES OF AMERICA

## Abstract

**Background:**

The recent human monkeypox (mpox) outbreak in 2022 has become a serious concern due to its rapid expansion to various non-endemic countries. There is limited information about the knowledge regarding mpox among the Bangladeshi population. Therefore, this study’s objectives were to: (i) determine the level of knowledge regarding mpox among undergraduate and post-graduate students in Bangladesh, and (ii) assess the determinants of knowledge regarding mpox among the study sample.

**Methods:**

An online-based cross-sectional study was conducted among 879 tertiary-level students from selected tertiary institutions (n = 13) in Bangladesh. The structured questionnaire consisted of two parts: (i) socio-demographic information and (ii) an assessment of knowledge regarding mpox. The Kruskal-Wallis test, Mann-Whitney test, and multivariable quantile regression model were employed.

**Results:**

The median age of the study participants was 23 years (IQR: 25–22). Low knowledge of mpox was found among study participants (20.7%, 23.2% and 56.1% had good, moderate and poor knowledge, respectively). The overall median knowledge score for mpox was 11 (IQR: 16–6). The median knowledge score of mpox significantly differed by participants’ gender, study major, and academic education about mpox. In the quantile regression analysis, the association between gender and mpox knowledge was observed at the 25^th^ (β **=** 1.343), 50^th^ (β **=** 2.00) and 75^th^ (β **=** 1.59) quantiles with females having more knowledge compared to males. The effects of study group were significant at 25^th^ (β **=** 1.746), 50^th^ (β **=** 1.5), 75^th^ (β **=** 1.361) and 90^th^ (β **=** 1.248) quantiles. Thus, those in medical or public health programs were likely to have more knowledge about mpox relative to those who were in non-medical related study groups. Students who received information about mpox during their education were more knowledgeable compared to those who had not, with statistical significance occurring at 10^th^ (β = 3.711), 25^th^ (β = 6.656), 50^th^ (β = 5.75), 75^th^ (β = 3.404) and 90^th^ (β = 2.592) quantiles.

**Conclusion:**

These findings imply that educational interventions about mpox should consider the gender dynamics and program of study among the students in Bangladesh.

## Introduction

This past century has seen rapid population growth, and greater levels of mobility. In addition, urban areas are undergoing an unprecedented expansion, transcending traditional boundaries. However, these changes have fostered an upsurge in the spread of zoonotic illnesses [1]. Zoonotic diseases can be viral, bacterial, or parasitic, and they can be transmitted to humans by food, drink, or direct contact [2]. The sheer variety of these ailments constitutes a substantial hazard to public health and must be closely monitored, examined, and prevented. Knowledge and awareness can play a crucial role in preventing the spread of the next global pandemic. Human monkeypox (mpox) is a zoonotic disease (part of the Orthopoxviral genus) characterized by clinical manifestations similar to those of smallpox [3]. Public health experts are concerned that the emergence of a new mpox outbreak could pose a new threat when the world is still dealing with the coronavirus disease 2019 (COVID-19) pandemic [4].

Several more cases have been recorded in areas where mpox is commonly found [3, 5, 6]. Since the start of May 2022, mpox cases have been identified in countries which are not typically susceptible to the virus. This has been a growing public health concern, considering the fact that this disease could be developed and disseminated through interactions between humans and animals [5]. Consequently, the WHO that confirmed the current global outbreak of mpox is a public health crisis emergency as of July 23, 2022 [7, 8]. The initial occurrence of mpox was documented in the Democratic Republic of the Congo (DRC) in 1970. However, other outbreaks of the disease have emerged and it has become prevalent in central and western African nations [9].

In contrast to the prevalence of the mpox virus in western and central Africa, a significant proportion of the confirmed cases with documented travel history have reported journeys to North America and Europe. The incidence of mpox cases has exhibited a substantial and notable escalation during the past two decades [10, 11]. As of March 7, 2023, a total of 81,000 confirmed cases and 55 fatalities had been recorded across 110 countries [12]. There is yet to be a confirmed case of mpox in Bangladesh, but nonetheless in May 2022, Bangladesh issued a health warning owing to the worldwide mpox outbreak [6].

The primary origin of mpox is attributed to wild animals, leading to potential transmission to humans through two distinct pathways: animal-to-human zoonotic transmission in endemic countries and human-to-human transmission in both endemic and non-endemic nations [14]. However, mpox is transferred mostly through respiratory droplets, bodily fluids, and close contact with infected animals’ skin lesions. The transmission of the disease to people can also occur through close physical proximity, direct face-to-face interaction, or intimate skin-to-skin contact [15, 16]. The incubation period of mpox varies between 4 and 21 days, during which patients may exhibit either asymptomatic or symptomatic conditions [17, 18]. Following the incubation phase, a significant proportion of individuals exhibit prodromal symptoms, including feelings of general discomfort, elevated body temperature, and enlargement of lymph nodes. The observed clinical manifestations of mpox include fever, discomfort in the back and head regions, the presence of a rash, general feelings of illness, and excessive fatigue [3]. Historically, the fatality rates associated with mpox cases is variable, ranging from 0% to 11% within the general population. In addition, it has been observed that young children are more susceptible to this disease [11, 19].

The question of mpox re-emergence remains unsolved, but the reason for a potentially alarming situation in the near future is due to the adaptability and the wide range of animal hosts of mpox [5, 7]. Furthermore, several risk factors such as lack of awareness, smallpox vaccination discontinuation, and increasing globalization may indicate that mpox will become a greater global public health concern in the future [8]. The recent growing incidence of mpox cases requires early detection, prompt response, management, and prevention. Although mpox has not yet been recorded in Bangladesh, the community must be vigilant to tackle an outbreak of mpox. Available data on the level of awareness and understanding of mpox within the Bangladeshi population is currently insufficient [9–12]. Therefore, the study was undertaken with the following aims: (i) to determine the level of knowledge regarding mpox among a sample of undergraduate and post-graduate students from selected tertiary institutions in Bangladesh, and (ii) to assess the determinants of knowledge regarding human mpox among the study population.

## Methods and materials

### Study design, participants and procedures

A cross-sectional study was conducted among tertiary-level students (i.e., undergraduate and post-graduate levels) who were studying medical science, public health, and other non-medical-related subjects at six private and seven public institutions in Bangladesh. The participants had to meet the following eligibility criteria: (i) be of age 18 years or older, (ii) currently be an enrolled student, and (iii) be a Bangladeshi citizen by birth. Eligibility criteria for the study were stated on the opening page of the survey questionnaire, and participants were instructed to consider these criteria before participating in the survey. Individuals under 18 years of age and those who were unwilling to participate were excluded from the study. In addition, any participant with missing information was excluded from the study. Missing values were handled using a list wise deletion technique, where a case is removed from an analysis if at least one of the specified variables had a missing value.

To determine sample size, we used the Cochran’s formula as a guide [13]. This formula allows us to calculate an ideal sample size (n) given a desired level of precision (e), desired confidence level (Z), and the estimated proportion of the attribute present in the population (p). The following assumptions were considered to calculate the sample size of this study: (i) Since there was no data on knowledge of mpox among Bangladeshi students when we designed our study protocol, a predicted prevalence of 50% was used (p = 0.5), (ii) 95% level of confidence (Z = 1.96), and (iii) 5% margin of error (e = 0.05). The calculation procedure is as follows: Sample size, $\text{n=}\frac{{\text{z}}^{\text{2}}\times \text{p}\times (1-\text{p})}{{\text{e}}^{2}}=\frac{\text{1}{\text{.96}}^{\text{2}}\times 0.5\times (1–0.5)}{\text{0}{\text{.05}}^{\text{2}}}=384.16\approx 385$.

Accordingly, the minimum required sample of 385 participants was calculated. To strengthen the external validity and generalizability of the study, we intended to include more participants than the calculated sample size [14]. Initially, a total of 927 responses were recorded, but 31 responses had missing information for certain observations and 17 respondents did not provide their consent. Thus, 879 participants made up the final study sample in this survey.

Recruitment was conducted using social networking sites (i.e. Facebook) and data was collected using Google Forms accessed via a survey link. There was no specific sampling technique; the purpose was to reach as many students as possible to collect the minimum calculated sample size or greater. The study team disseminated the survey link to different public Facebook pages or groups of the selected institutions, where university or medical students connect virtually. The data for this study was gathered between 20 May 2023 and 10 July 2023.

A pilot study was carried out to assess the accessibility and consistency of the questionnaire before its administration. Perneger et al. [15] suggested a default sample size of 30 participants for pre-testing the questionnaire to give sufficient power to detect fairly typical problems. In order to pre-test the questionnaire, 30 university students were randomly selected from the lead author’s institution. During the pilot survey, no significant problems with the questionnaire were brought up by the respondents. Filling in the questionnaire took on average 8 to 10 minutes to complete. The English version of the questionnaire was used, since English is the medium of instruction in universities and medical colleges in Bangladesh, there were no linguistic barriers with the survey items from the respondents. The results of the pilot study were not included in the final analysis. To minimize the possibility of double participation, pilot-survey respondents were requested not to take part in the final study, even if they received the survey link.

### Study variables and measures

Respondents’ demographic information such as gender, age, study major, study level, institution type, residency, parent’s education and occupation, and whether they had ever heard about mpox were collected as independent variables.

A set of 21 questions was used to evaluate the respondents’ knowledge of mpox [16], which is the outcome variable of this study. The knowledge items are as follows: Five questions assessed knowledge of the source, definition, and incubation period; two questions assessed the transmission mechanism of mpox; seven questions assessed the signs and symptoms; five questions assessed the preventative measures; and two questions assessed the treatment methods. The participants were to choose one of three possible answers to each of the 21 questions: "Yes," "No," or "I don’t know." To reduce the possibility of selecting the correct answer by chance, the option “I don’t know” was used. The reliability of this scale was good (Cronbach’s alpha = 0.74).

One point was allocated for the correct answer, and zero points were allocated for the incorrect answer. The overall knowledge score ranged from 0 to 21. A higher score indicated higher knowledge regarding mpox. The knowledge score (a continuous variable) was used as outcome variable in this study to assess factors that predict knowledge score of mpox.

To better understand respondent’s overall knowledge about mpox, a percentage of knowledge score was c0mputed and classified as good, moderate, or poor. The percentage (%) knowledge score was calculated using the following formula [16]:

$$\text{\%}\;\text{of}\;\text{knowledge}\;\text{score=}\frac{\text{Number}\;\text{of}\;\text{questions}\;\text{correctly}\;\text{answered}\;\text{by}\;\text{the}\;\text{participant}}{\text{21}}\times 100.$$

Several previous studies used modified Bloom’s cut-off range to categorize the level of overall knowledge (i.e., good vs. moderate vs. poor) on a specific disease [16, 17]. Thus, participants’ overall knowledge of mpox was rated as "good" if they scored between 80% and 100% (i.e., 17 to 21 points), "moderate" if they scored between 60% and 79% (i.e., 16.5 and 13 points), and "poor" if they scored below 60% (<13 points) [16, 17]. This classification was used to illustrate participants’ general knowledge of mpox, not for predictor analysis.

### Statistical approach

Both enumerative statistics (such as frequencies, percentages, median, etc.) and inferential statistics (such as regression analysis) were employed to analyze the data. Inferential statistics employ a sample to draw reasonable conclusions about the population if the sample was selected using random and unbiased sampling methods (i.e., probability sampling) [18]. However, non-probability sampling technique was used in our study because of its feasibility, ease of usage, and low cost [19]. We performed an inferential analysis of our survey data to make a fair conclusion about the population from a sample by assuming that our study sample was representative of the population. Previous studies have adopted a similar approach [9, 20–24].

A Breusch-Pagan/Cook-Weisberg test for heteroscedasticity was performed and the test observed heteroscedasticity (Chi-square = 2.06, p = 0.025). In addition, a Shapiro-wilk W test showed that the distribution of knowledge scores departed significantly from normality (W = 0.871, p < 0.001); therefore, a non-parametric test was used. A Kruskal-Wallis test and Mann-Whitney test were utilized to compare knowledge scores across the explanatory variables. In this comparison, data were presented as the median and interquartile range (IQR).

Quantile regression, an extension of linear regression, is often suitable to apply when the assumptions of ordinary least square (OLS) regression such as homoscedasticity and normality are not fulfilled [25–27]. Since our data violated the potential criteria of OLS regression, multiple quantile regression models were fitted to identify the predictors of knowledge regarding mpox. Multivariable quantile regression model was used to display the distribution of knowledge scores across the explanatory variables in different quantiles (10^th^, 25^th^, 50^th^, 75^th^ and 90^th^ quantiles). The goodness of fit in quantile regression was checked using Pseudo R^2^ (Pseudo R^2^ for 10th, 25^th^, 50^th^, 75^th^ and 90th quantiles model was 0.0777, 0.1411, 0.0916, 0.0842 and 0.0717, respectively). Regression coefficient (β) and 95% confidence interval (CI) were estimated for the selected quantiles of the knowledge scores based on 500 bootstrap replications. Five hundred bootstrap replications were performed to obtain unbiased estimates and representativeness of the population [28, 29]. A p-value of <0.05 was considered statistically significant throughout the analysis. All statistical analysis was performed by STATA (BE version 17.0, StataCorp, College Station, TX, USA).

### Ethics

The study protocol was reviewed and approved by the Institutional Ethical Committee (IEC) of Patuakhali Science and Technology University, Bangladesh (reference number: PSTU/IEC/2022/51). Written informed consent was obtained from all participants. Anonymity and confidentiality of participant information was assured.

## Results

### Sample characteristics

A total of 879 students from the selected 13 tertiary institutions participated in this study. The median age of the study population was 23 years (IQR: 25–22). The majority of participants were aged 22 to 24 years (47.9%). More than half of the participants were female (53.2%), and the rest of them were male (46.8%). Nearly 40% of the participants (39.7%) were studying medical science or public health. Three-quarters of the participants (74.2%) were studying at an undergraduate level. One third of the participants (33.1%) were studying in private institutions and the rest, up to two-thirds (66.9%) of the participants were studying in public institutions. The majority of the participants resided in urban areas (73.0%). More than one-third (34.2%) of the participants’ mothers had an education level of honors or above. The majority of the participants’ mothers were housewives (77.4%). More than half of the participants’ fathers had an education level of honors or above (58.8%). Over one-third of the respondents’ fathers were businessmen (35.0%). Around two-thirds of the participants (66.8%) had received information regarding human mpox during academic education. The detailed socio-demographic features of study participants are summarized in Table 1.

**Table 1:** The proportion of respondents based on their socio-demographic characteristics (N = 879).

Variables	Frequency	Percentage
**Gender**		
Male	411	46.8
Female	468	53.2
**Age** (in years)		
18–21	198	22.5
22–24	421	47.9
25 and above	260	29.6
**Study major**		
Medical or public health	349	39.7
Non-medical science	530	60.3
**Study level**		
Undergraduate	652	74.2
Post-graduate	227	25.8
**Institution type**		
Private	291	33.1
Public	588	66.9
**Residency**		
Rural area	237	27.0
Urban area	642	73.0
**Mothers’ education level**		
Illiterate	17	1.9
Primary	74	8.4
Secondary	209	23.8
Higher secondary	278	31.6
Honors and above	301	34.2
**Mother’s occupation**		
Housewife	680	77.4
Employed	175	19.9
Others	24	2.7
**Father’s education level**		
Illiterate	10	1.1
Primary	55	6.3
Secondary	110	12.5
Higher secondary	187	21.3
Honors and above	517	58.8
**Father’s occupation**		
Governmental job	289	32.9
Private job	141	16.0
Business	308	35.0
Others	141	16.0
**Received information of mpox during education**		
Yes	587	66.8
No	292	33.2

### Knowledge of mpox among study participants

The assessment of participants’ knowledge regarding mpox is summarized in Table 2. The majority of respondents (77.8%) were aware that mpox is caused by a virus. Approximately one-third of participants (33.2% to 37.2%) reported that they did not know the transmission mechanism of mpox. More than half of the respondents (52.9%) were aware that flu-like syndrome is one of the early signs or symptoms of mpox, while 38.1% of them stated they did not know about the early signs or symptoms of the disease. Around half of the respondents (47.2% and 49.9%, respectively) indicated they did not know that vesicles and pustules on the skin are one of the signs or symptoms of mpox. Nearly one-third to half of the respondents (30.5% to 49.1%) reported that they did not know about the different preventive measures for mpox (15 to 19 number items). More than half of the participants (53.5%) wrongly answered that mpox can be treated with the available antiviral medications (Table 2).

**Table 2:** Responses to knowledge items regarding human mpox by study participants (N = 879).

Questions	Response, n (%)		
	Yes	No	Don’t Know
*Source*, *definition*, *and incubation time*			
1. Mpox is a viral disease infection.	684 (77.8)	23 (2.6)	172 (19.6)
2. Mpox is a bacterial disease infection.	189 (21.5)	430 (48.9)	260 (29.6)
3. Mpox occurs in primarily in tropical rainforest areas of Africa and is occasionally exported to another region.	501 (57.0)	58 (6.6)	320 (36.4)
4. Mpox and smallpox have similar signs and symptoms.	407 (46.3)	147 (16.7)	325 (37.0)
5. The interval from infection to onset of symptoms is usually from 6 to 13 days but can range from 5 to 21 days.	344 (39.1)	58 (6.6)	477 (54.3)
*Route of transmission*			
6. Mpox is easily transmitted animal-to-human, through direct contact with the blood, bodily fluid, cutaneous or mucosal lesions of infected animal or eating insufficiently cooked meat from an infected animal.	461 (52.4)	91 (10.4)	327 (37.2)
7. Mpox is easily transmitted human -to-human through close contact with respiratory secretions, skin lesions of the infected person, or contaminated objectives.	528 (60.1)	59 (6.7)	292 (33.2)
*Signs and symptoms*			
8. Flu-like syndrome is one of the early signs or symptoms of human mpox.	465 (52.9)	79 (9.0)	335 (38.1)
9. Skin rashes usually begin within 1–3 days of fever are one of the signs or symptoms of human mpox.	496 (56.4)	40 (4.6)	342 (39.0)
10. Papules on the skin are one of the signs or symptoms of human mpox.	406 (46.2)	40 (4.6)	343 (39.0)
11. Vesicles on the skin are one of the signs or symptoms of human mpox.	368 (41.9)	96 (10.9)	416 (47.2)
12. Pustules on the skin are one of the signs or symptoms of human mpox.	357 (40.6)	83 (9.4)	439 (49.9)
13. Lymphadenopathy (swollen lymph nodes) is one clinical sign or symptom that could be used to differentiate mpox and smallpox cases.	375 (42.7)	53 (6.0)	451 (51.3)
14. Fever, Exhaustion, back and muscle ache and intense headache are the signs or symptoms of human mpox.	528 (60.1)	48 (5.5)	303 (34.5)
*Preventive measures*			
15. Mpox could be prevented by cooking meat properly.	303 (34.5)	144 (16.4)	432 (49.1)
16. Avoiding contact with any objectives that have been in contact with sick animals can prevent spread of disease.	508 (57.8)	70 (8.0)	301 (34.2)
17. Avoiding contact with any person that has a rash can prevent the spread of disease.	494 (56.2)	92 (10.5)	293 (33.3)
18. Avoiding contact with any objective that has been in contact with sick people can prevent spreading disease.	492 (56.0)	75 (8.5)	312 (35.5)
19. Reporting symptoms of mpox to local health authorities is important to prevent further disease transmission.	572 (65.1)	39 (4.4)	268 (30.5)
*Treatment*			
20. Mpox is usually a self-limited disease with the symptoms lasting from 2 to 4 weeks.	302 (34.3)	117 (13.3)	460 (52.3)
21. Mpox can be treated with the available antiviral medications.	408 (46.4)	97 (11.0)	374 (42.5)

**Fig 1:**
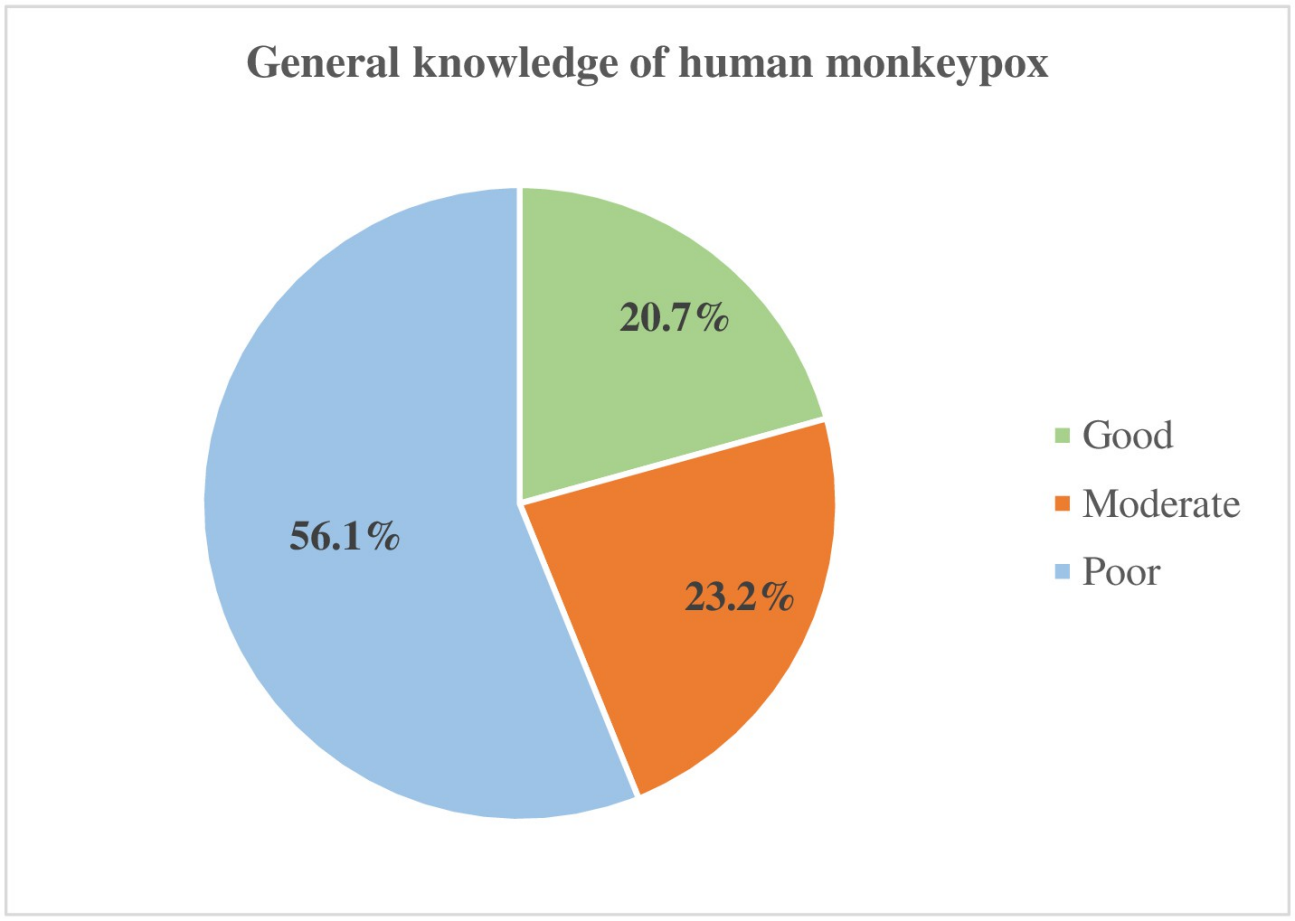
The proportions of respondents by their level of knowledge based on the modified Bloom’s categorization of knowledge levels (N = 879).

Based on the modified Bloom’s cut-off point, 20.7% of the participants had a good level of knowledge about mpox, 23.2% had moderate knowledge and 56.1% had poor knowledge (Fig 1).

Overall, the median knowledge score for mpox was 11 out of 21 (IQR: 16–6). The median knowledge score of mpox significantly differed in function to participants’ gender (p = 0.008) and study major (p < 0.001), and participants who received information regarding mpox during academic education (p < 0.001). The median knowledge score of mpox was not significantly varied by participants’ age, study level, institution type, residency, and parents education and occupation (p > 0.05) (see Table 3).

**Table 3:** Differences in mpox-related median knowledge score by the respondents’ socio-demographic characteristics.

Variables	Mann-Whitney Test		*Kruskal—Wallis Test*		P value
	Median	IQR	Median	IQR	
**Gender**					**0.008**
Male	11	15–5			
Female	12	16–6.5			
**Age** (in years)					0.180
18–21			11	15–4	
22–24			11	15–6	
25 and above			12	17–6	
**Study major**					**<0.001**
Medical or public health	13	16–8			
Non-medical science	11	15–5			
**Study level**					0.062
Under graduate	11	15–6			
Post-graduate	12	16–6			
**Institution type**					0.126
Private	12	16–6			
Public	11	15–6			
**Residency**					0.591
Rural area	12	16–5			
Urban area	11	16–6			
**Mothers’ education level**					0.135
Illiterate			11	16–4	
Primary			13	18–6	
Secondary			12	16–5	
Higher secondary			11	15–5	
Honors and above			12	16–7	
**Mother’s occupation**					0.409
Housewife			12	16–6	
Employed			11	16–7	
Others			8.5	14.5–5	
**Father’s education level**					0.575
Illiterate			8	14–3	
Primary			12	16–5	
Secondary			11	16–5	
Higher secondary			11	15–6	
Honors and above			12	16–6	
**Father’s occupation**					0.496
Governmental job			12	16–6	
Private job			11	16–6	
Business			11	15–5.5	
Others			12	16–5	
**Received information of mpox during education**					**<0.001**
Yes	13	17–8			
No	8	13.5–1			

Note: IQR = Interquartile range. Bolded values indicate statistically significance.

Table 4 presents the results of the multivariable quantile regression analysis. In the multivariate analysis, the effect of gender on mpox knowledge was observed at the 25^th^ (β **=** 1.343; 95%CI = 0.19, 2.49), 50^th^ (β **=** 2; 95%CI = 0.77, 3.22) and 75^th^ (β **=** 1.59; 95%CI = 0.46, 2.72) quantiles with females having more knowledge compared to males. Nonetheless, the effects of study group were significant at 25^th^ (β **=** 1.746; 95%CI = 0.58, 2.90), 50^th^ (β **=** 1.5; 95%CI = 0.09, 2.90), 75^th^ (β **=** 1.361; 95%CI = 0.29, 2.43) and 90^th^ (β **=** 1.248; 95%CI = 0.41, 2.08) quantiles. Those in medical or public health programs were likely to have more knowledge about mpox relative to those who were in non-medical groups. In terms of study level, there was an effect at the 75^th^ quantile (β **=** 1.617; 95%CI = 0.13, 3.10) with those in post-graduate level having more knowledge about mpox.

**Table 4:** Multivariable quantile regression analysis showing the predictors of knowledge regarding human mpox among study participants.

Variables	10^th^ quantile	25^th^ quantile	50^th^ quantile	75^th^ quantile	90^th^ quantile
	β (95%CI)	β (95%CI)	β (95%CI)	β (95%CI)	β (95%CI)
**Gender**					
Male	Ref.		Ref.		
Female	0.678 (-0.43, 1.78)	1.343 (0.19, 2.49)*	2 (0.77, 3.22)**	1.59 (0.46, 2.72)**	0.941 (-0.035, 1.91)
**Age** (in years)					
18–21	Ref.		Ref.		
22–24	0.525 (-0.89, 1.94)	0.447 (-1.12, 2.025)	-0.875 (-2.51, 0.76)	-1.085 (-2.36, 0.19)	-0.171 (-1.37, 1.02)
25 and above	-0.050 (-1.89, 1.78)	0.701 (-1.22, 2.63)	0.5625 (-1.67, 2.79)	-0.404 (-2.10, 1.29)	-0.312 (-1.67, 1.05)
**Study group**					
Medical or public health	1.203 (-0.28, 2.68)	1.746 (0.58, 2.90)**	1.5 (0.09, 2.90)*	1.361 (0.29, 2.43)*	1.248 (0.41, 2.08)**
Non-medical science	Ref.		Ref.		
**Study level**					
Under graduate	Ref.		Ref.		
Post-graduate	0.389 (-1.22, 2.00)	1.179 (-0.35, 2.71)	0.5 (-1.36, 2.36)	1.617 (0.13, 3.10)*	0.855 (-0.39, 2.09)
**Institution type**					
Private	Ref.		Ref.		
Public	0.305 (-1.06, 1.67)	-0.164 (-1.44, 1.11)	-0.812 (-2.23, 0.60)	-1.382 (-2.42, -0.34)**	-1.402 (-2.41, -0.39)**
**Residency**					
Rural area	Ref.		Ref.		
Urban area	0.423 (-0.99, 1.84)	0.059 (-1.47, 1.6)	-0.625(-2.11, 0.86)	-0.893 (-2.21, 0.42)	0.135 (-0.95, 1.22)
**Mothers’ education level**					
Illiterate	Ref.		Ref.		
Primary	2.661(-4.47, 9.79)	1.029 (-5.15, 7.21)	0.375 (-4.41, 5.16)	-0.468 (-5.80, 4.86)	-0.058 (-3.87, 3.75)
Secondary	0.050 (-7.27, 7.37)	-0.925 (-7.16, 5.30)	-1.5 (-6.36, 3.36)	-3.319 (-8.45, 1.81)	-2.217 (-6.18, 1.75)
Higher secondary	0.644 (-6.85, 8.14)	-0.761 (-6.92, 5.40)	-2.625 (-7.58, 2.33)	-4.702 (-9.99, 0.59)	-3.158 (-7.29, 0.98)
Honors and above	1.627 (-5.86, 9.12)	-0.253 (-6.43, 5.92)	-2.312 (-7.59, 2.97)	-3.914 (-9.39, 1.56)	-1.909 (-6.03, 2.21)
**Mother’s occupation**					
Housewife	Ref.		Ref.		
Employed	0.559 (-1.17, 2.29)	-0.104(-1.62, 1.42)	-0.375 (-2.31, 1.56)	-0.787 (-2.50, 0.93)	0.217 (-0.85, 1.28)
Others	-0.254 (-3.58, 3.08)	-2.910(-5.58, -0.24)*	-3.18(-6.97, 0.597)	-2.446 (-5.27, 0.38)	-3.525 (-6.65, -0.40)*
**Father’s education level**					
Illiterate	Ref.		Ref.		
Primary	-3.542 (-10.92, 3.84)	-0.074(-6.67, 6.52)	3.25 (-2.72, 9.22)	3.361 (-3.59, 10.31)	1.226 (-4.61, 7.06)
Secondary	-2.016 (-9.18, 5.15)	0.283 (-5.89, 6.45)	3.5(-2.70, 9.70)	2.425 (-4.57, 9.43)	1.570 (-4.52, 7.66)
Higher secondary	-1.237 (-8.75, 6.28)	0.582 (-5.89, 7.05)	3.5(-2.74, 9.74)	2.957(-4.10, 10.02)	1.719 (-4.23, 7.67)
Honors and above	-1.949 (-9.51, 5.61)	0.985 (-5.33, 7.30)	5.062(-1.11, 11.23)	4.042 (-2.99, 11.07)	1.276 (-4.64, 7.19)
**Father’s occupation**					
Governmental job	Ref.		Ref.		
Private job	0.101 (-1.8, 2.03)	-0.402 (-1.93, 1.12)	-1.5(-3.72, 0.72)	-0.510 (-2.17, 1.15)	-0.542 (-1.67, 0.58)
Business	0.118 (-1.38, 1.62)	0.313 (-1.17, 1.79)	-0.68(-2.32, 0.94)	-1.31 (-2.70, 0.06)	-0.429 (-1.65, 0.79)
Others	-0.322 (-2.09, 1.45)	1.223(-1.19, 3.63)	0.875(-1.09, 2.84)	-0.382 (-2.01, 1.25)	-0.787 (-2.19, 0.61)
**Received information of human mpox during education**					
Yes	3.711 (2.27, 5.15)***	6.656(5.37, 7.94)****	5.75(4.03, 7.46)***	3.404 (2.26, 4.54)***	2.592 (1.41, 3.77)***
No	Ref.		Ref.		

Note: β = regression coefficient, CI = Confidence Interval and Ref.: reference category. Asterisk values indicate statistically significant (p value <0.05).

* p value <0.05,

** p value <0.01 and

*** p value <0.001.

With institution type, it was observed that participants from public institutions had low knowledge compared to those at the private institutions with significant results occurring at 75^th^ (β = -1.382; 95%CI = -2.42, -0.34) and 90^th^ quantiles (β = -1.402; 95%CI = —2.41, -0.39). The results further showed that students who received information of mpox during their education were more knowledgeable compared to those who had not, with statistical significance occurring at 10^th^ (β = 3.711; 95%CI = 2.27, 5.15), 25^th^ (β = 6.656; 95%CI = 5.37, 7.94), 50^th^ (β = 5.75; 95%CI = 4.03, 7.46), 75^th^ (β = 3.404;95%CI = 2.26, 4.54) and 90^th^ (β = 2.592; 95%CI = 1.41, 3.77) quantiles (Table 4).

## Discussion

This study assessed the level of knowledge regarding mpox among tertiary-level students in Bangladesh. It was found that more than half (56.1%) of the students had poor knowledge and only one-fifth (20.7%) had good knowledge. The findings on the low knowledge score is consistent with the findings in several previous studies in Jordan [30], Iraq [31], Kuwait [32], Indonesia [33] and Pakistan [34]. Specifically, the study by Kumar et al. [34] showed that only 6.3% had good knowledge on mpox transmission. Nonetheless, our findings are different from what was found in a Nigerian study by Al-Mustapha et al. [35] where 58.7% of participants had a good knowledge of the incubation period, symptoms, route of transmission, and preventive practices on mpox. The possible variation in the study findings could be differences in the study population, locations and periods. The Nigerian study was conducted among the general population and the general population in Nigeria was more knowledgeable about mpox (58.7%) than what we found among Bangladeshi students in tertiary institutions (20.7%). The plausible explanation could be that the public awareness campaigns that were conducted during an mpox outbreak in Nigeria led to a more informed population.

At the multivariate level the quantile regression analysis showed statistically significant results in terms of gender, study group, study level, institution type and exposure to mpox education. With gender, our results showed significant variations in knowledge at various quantiles of mpox knowledge distribution. Females had statistically significant higher knowledge compared to males at 25^th^, 50^th^ and 75^th^ quantiles. The effect size was moderate at the 25^th^ percentile and increased at the 50^th^ percentile suggesting that gender gaps in the knowledge score is more pronounced at the 50^th^ percentile among males and females. This finding suggests that gender differences in knowledge score are not the same across the knowledge spectrum. This finding is consistent with previous a study [16] that also found that female students had a higher knowledge score of mpox compared to their male counterparts. The possible reason for this finding is that females are usually more concerned about health conditions compared to males. Existing research has consistently demonstrated that, as a general trend, females exhibit higher levels of health literacy when compared to males [36]. This necessitates the importance of gender specific interventions in mpox knowledge interventions paying more attention to males.

In terms of study group, our results showed significant variations in knowledge at various quantiles of mpox knowledge distribution. For example, this was significant at 25^th^, 50^th^, 75^th^, and 90^th^ quantiles. The strongest effect was observed at the 25^th^ quintile suggesting that differences in terms of study group and mpox knowledge score are more evident in those with low knowledge scores. Specifically, those in medical or public health programs were more likely to have knowledge about mpox relative to those who were in non-medical groups. This is similar to the findings obtained by Jairoun et al. [16] among university students in United Arab Emirates and Hassan et al. [6] among medical doctors in Bangladesh. There are several pathways to explain this association. First, students in health sciences have basic knowledge on diseases transmission from their previous education [37]. Second, in their present education, some of them are introduced to courses such as emerging and reemerging diseases, and epidemiology in general.

Relatedly, the study showed that students in public institutions have lower knowledge about mpox compared to those at the private institutions with significant effects at 75^th^ and 90^th^ quantiles suggesting differences widens at higher quantiles of mpox knowledge distribution. These differences could be attributed to differences in access to educational resources, curriculum type and emphasis on public health threats and pandemics. This underscores the need to increase awareness of mpox among students in public universities.

The findings also revealed that students who received information about mpox as part of their education were consistently more knowledgeable compared to those who had not, with statistically significant effects observed across the 10th, 25^th^, 50^th^, 75^th^, and 90^th^ quantiles. The effect was most pronounced at the 25^th^ quantile, indicating that among students with lower baseline knowledge, those who received mpox-related education had substantially higher knowledge levels. Even at the 10^th^ quantile where knowledge levels were generally the lowest, students who had been exposed to mpox information had significantly higher knowledge. As explained by Jairoun et al. [16] the resurgence of mpox globally has underscored the necessity for media outlets to prioritize risk communication efforts, particularly in advocating for zoonotic diseases. A consistent stream of daily updates can play a crucial role in enhancing the general public’s understanding and awareness of mpox. Consequently, it is plausible that individuals exposed to education on mpox attain higher knowledge scores due to their enhanced accessibility to precise and comprehensive information about mpox. Further, Ibrahim and Zaghamir’s [38] study underscores the importance of educational interventions in enhancing knowledge and fostering positive attitudes towards infectious diseases such as mpox.

### Strength and limitations

This study has various strengths and limitations. First, the study employed a cross-sectional survey, therefore the findings could only be viewed in terms of association but not causal inferences. Second, since the study used non-probability sampling to enroll participants, the study findings could not generalize to the entire student population in Bangladesh. Third, the study, conducted among educated participants with internet access, may only reflect educated youths in Bangladesh, particularly university/college students. Selection bias was also possible since this was an online survey therefore, only those who showed interest and saw it convenient participated. Despite these limitations, to the best of our knowledge this is one of the first studies to assess the knowledge level of mpox among undergraduate and post-graduate students in Bangladesh. A strength of this study was that the study was not limited to only one university but included different types of university students from different backgrounds within Bangladesh.

## Conclusion

In conclusion, the study revealed relatively poor overall knowledge status on mpox. There was also poor knowledge on preventive measures of mpox. Gender, study major, institution type and information during academic education were associated with knowledge score on mpox. Since there is low knowledge on mpox, it is important to increase awareness and knowledge among university students in Bangladesh to help them prepare for a possible future mpox outbreak. These findings imply that educational interventions about mpox should consider the gender dynamics and program of study among the students. Further studies employing different study designs, including intervention study designs, are required especially for students in public institutions.

## Supporting information

S1 Data (XLS)
